# Identification of miRNA–mRNA regulatory network associated with the glutamatergic system in post-traumatic epilepsy rats

**DOI:** 10.3389/fneur.2022.1102672

**Published:** 2022-12-23

**Authors:** Xiaoyuan Zhang, Yixun Ma, Fengjuan Zhou, Mengzhou Zhang, Dong Zhao, Xu Wang, Tiantong Yang, Jun Ma

**Affiliations:** ^1^Key Laboratory of Evidence Science, Institute of Evidence Law and Forensic Science, China University of Political Science and Law, Ministry of Education, Beijing, China; ^2^Collaborative Innovation Center of Judicial Civilization, Beijing, China; ^3^College of Biological Science, China Agricultural University, Beijing, China; ^4^Chinese Institute for Brain Research, Beijing, China; ^5^Department of Radiology, Chui Yang Liu Hospital Affiliated to Tsinghua University, Beijing, China

**Keywords:** post-traumatic epilepsy, glutamatergic system, RNA sequencing, miRNA, mRNA

## Abstract

**Background:**

Glutamate is one of the most important excitatory neurotransmitters in the mammalian brain and is involved in a variety of neurological disorders. Increasing evidence also shows that microRNA (miRNA) and mRNA pairs are engaged in a variety of pathophysiological processes. However, the miRNA and mRNA pairs that affect the glutamatergic system in post-traumatic epilepsy (PTE) remain unknown.

**Methods:**

PTE rats were induced by injecting 0.1 mol/L, 1 μL/min FeCl_2_ solution. Behavioral scores and EEG monitoring were used to evaluate whether PTE was successfully induced. RNA-seq was used to obtain mRNA and miRNA expression profiles. Bioinformatics analysis was performed to screen differentially expressed mRNAs and miRNAs associated with the glutamatergic system and then predict miRNA–mRNA interaction pairs. Real-time quantitative reverse transcription PCR was used to further validate the expression of the differential miRNAs and mRNAs. The microRNA–mRNA was subject to the Pearson correlation analysis.

**Results:**

Eight of the 91 differentially expressed mRNAs were associated with the glutamatergic system, of which six were upregulated and two were downregulated. Forty miRNAs were significantly differentially expressed, with 14 upregulated and 26 downregulated genes. The predicted miRNA–mRNA interaction network shows that five of the eight differentially expressed mRNAs associated with the glutamatergic system were targeted by multiple miRNAs, including *Slc17a6, Mef2c, Fyn, Slc25a22*, and *Shank2*, while the remaining three mRNAs were not targeted by any miRNAs. Of the 40 differentially expressed miRNAs, seven miRNAs were found to have multiple target mRNAs associated with the glutamatergic system. Real-time quantitative reverse transcription PCR validation and Pearson correlation analysis were performed on these seven targeted miRNAs—*Slc17a6, Mef2c, Fyn, Slc25a22*, and *Shank2*—and six additional miRNAs selected from the literature. Real-time quantitative reverse transcription PCR showed that the expression levels of the mRNAs and miRNAs agreed with the predictions in the study. Among them, the miR-98-5p–Slc17a6, miR-335-5p–Slc17a6, miR-30e-5p–Slc17a6, miR-1224–Slc25a22, and miR-211-5p–Slc25a22 pairs were verified to have negative correlations.

**Conclusions:**

Our results indicate that miRNA–mRNA interaction pairs associated with the glutamatergic system are involved in the development of PTE and have potential as diagnostic biomarkers and therapeutic targets for PTE.

## 1. Introduction

Post-traumatic epilepsy (PTE) is the most devastating sequela of traumatic brain injury (TBI), and seriously affects the quality of life of the injured (1, 2). PTE patients not only suffer from recurrent spontaneous seizures, but also from memory and cognitive loss, depression, and numerous other adverse effects (3). These signs and symptoms are caused by neuronal damage. An imbalance in the regulatory mechanisms of neuronal excitation and inhibition leads to excessive synchronous firing of neurons. In this process, glutamate and other neurotransmitters are widely involved in the regulation process between neuronal excitation and inhibition by activating corresponding receptors (4). The occurrence of PTE is related to excessive release of glutamate. After TBI, glutamate release is increased, and the regulatory mechanisms of neuronal excitation and inhibition are unbalanced, which further induces neuronal cell injury, death, and dysfunction, and finally induces PTE (5–7). Although more than 20 antiseizure medications are used in the clinical treatment of PTE, ~30% of patients with PTE still experience drug-resistant seizures (8). Understanding the molecular mechanisms of PTE will help to identify new therapeutic strategies for PTE. The imbalance of the neuronal regulatory mechanism triggered by glutamate is an essential reason for the development of PTE. Therefore, it is necessary to study the molecular mechanism of glutamate related to PTE to identify new therapeutic strategies.

Abnormal expression of glutamatergic mRNA has been observed after TBI. γ-aminobutyric acid (GABA) is the main inhibitory neurotransmitter in the brain, which is formed by the decarboxylation of glutamic acid by glutamic acid decarboxylase (GAD). In the fluid-percussion injury model, GABA_A_ receptor subunit mRNAs show abnormal expression in different regions of the rat brain. This may result in diminished GABA_A_ receptor-mediated inhibition (9). However, the development of PTE will be affected after the normal level of glutamatergic mRNA expression is restored. For example, SLC1A2 expression was downregulated after TBI, and PTE symptoms were alleviated after normalizing SLC1A2 expression with ceftriaxone (10). Because TBI directly induces PTE, the abnormal expression of glutamatergic mRNA after TBI may be one of the molecular mechanisms of PTE. In addition to mRNA, microRNAs (miRNA) are also an important component of the molecular mechanism of TBI. As potential diagnostic and predictive biomarkers, miRNAs do not encode proteins, but can regulate the expression of target mRNAs at the post-transcriptional level and subsequently affect the expression level of proteins (11). During TBI and epilepsy, miRNAs are involved in neuronal cell apoptosis, the inflammatory response, synaptic remodeling, abnormal conduction pathway formation, and other neuronal cell damage and repair. For example, several studies have confirmed the abnormal expression of miR-21 and miR-92a after TBI (12–14), and miR-139-5p and others play a key role in various pathophysiological mechanisms of epilepsy (15–17). However, the aforementioned differences in mRNA and miRNA expression have not been verified in the PTE model, so the glutamate-related molecular mechanisms of PTE are still unknown.

The mRNA–miRNA regulatory network plays an important role in the study of several disease mechanisms. For example, a study on mesial temporal lobe epilepsy (mTLE) identified key mRNAs and miRNAs involved in mTLE by establishing and analyzing the mRNA–miRNA regulatory network. miR-27a-3p was identified as a potential diagnostic biomarker for mTLE (18). Some studies have established a circular RNA (circRNA)–miRNA–mRNA regulatory network to analyze the interactions between circRNA, miRNA, and mRNA in the control cortical shock (CCI) model, to understand the molecular mechanism of TBI and screen potential therapeutic targets (19). Considering the important role of the mRNA–miRNA regulatory network in the study of disease mechanisms, it is necessary to uncover the regulatory relationship of miRNA–glutamatergic mRNA to understand the pathogenic mechanisms of PTE and glutamatergic association.

To understand the differential expression of glutamatergic mRNA and miRNA during the development of PTE and the possible targeting relationship between them, and to reveal the glutamatergic-related molecular mechanism of the development of PTE, we used transcriptome sequencing technology to identify differentially expressed miRNA and glutamatergic mRNA in the rat model of PTE. Bioinformatics methods were used to draw the miRNA–mRNA targeting network, and real-time quantitative reverse transcription PCR was used to verify the negative targeting relationship between miRNA and mRNA. Our results suggest that miRNA–mRNA interaction pairs in the glutamate system are involved in the development of PTE, and have potential as diagnostic biomarkers and therapeutic targets for PTE.

## 2. Materials and methods

### 2.1. Ethics statement

The Animal Research Ethics Committee of the Institute of Evidence Science, China University of Political Science and Law, approved the animal study (#2019012). All the animal experiments and procedures were performed following the guidelines of the Weatherall Committee and the National Centre for the Replacement, Refinement, and Reduction of Animals in Research (NC3Rs; London, UK).

### 2.2. Animals and sample preparation

A total of 15 male Sprague-Dawley rats (7–8-months old, weighing 210–230 g) were purchased from Beijing Laboratory Animal Research Center (<city>Beijing </city>, China) and housed with a 12-h light/dark cycle with free access to water and food. After a 72-h acclimatization period, the rats were randomly divided into sham and PTE groups (*n* = 6/group). A FeCl_2_-induced PTE model was established as previously described (20). Briefly, the rats were anesthetized with 2% pentobarbital sodium (40 mg/kg) *via* intraperitoneal injection and placed in a stereotactic device (Nanjing Medease Science and Technology, Jiangsu, China). The electrodes were fixed to the rat frontal and occipital lobes and connected with a biosignal acquisition system (Shanghai creaform3d information, Shanghai, China) for EEG collection. The rats in the PTE group were injected with 10 μl FeCl_2_ (0.4 mol/L, 1 μl/min) at the right frontal cortex (2.0 mm anterior to bregma, 3.0 mm from midline, and 2.0 mm depth) using a microinjector. The sham group underwent the same procedure except for the injection. The rats were killed on the 13th day after the operation. The brain tissue samples surrounding the injection sites were collected and stored at −80°C for further experiments.

### 2.3. Behavioral assessment and electroencephalography monitoring

The severity of seizures was assessed at 1 h before the operation, 1 h after the operation, and once daily thereafter for 30 consecutive days in accordance with the Racine's scale (21): 0, no abnormality; 1, staring; 2, head nodding or wet-dog shaking with or without facial tics; 3, unilateral forelimb clonus; 4, bilateral forelimb clonus and continuous head nodding; 5, exacerbated bilateral forelimb clonus, loss of balance and falling, or generalized tonic–clonic seizures.

A 1-h EEG, consisting of the first 15-min and 10-min blocks at 5-min intervals, was recorded at 1 h before surgery and at 1, 7, and 30 days after surgery.

The criteria for a successful PTE model include: (1) Racine's score > 4, (2) sharp waves or spike waves on EEG, and (3) paroxysmal or continuous abnormal discharges on EEG.

### 2.4. Total RNA isolation, library construction, and sequencing

Total RNA was isolated from frontal lobe brain tissues adjacent to the FeCl_2_ injection region of rats using TRIzol™ reagent (#15596026, Thermo Fisher Scientific, Waltham, MA, USA) following the manufacturer's protocol. The RNA quality was monitored on 1% agarose gels. The purity and quantity of RNA were determined using a NanoPhotometer^®^ device (Implen, Camarillo, CA, USA) and a Qubit^®^ 2.0 kit, respectively. The RNA integrity was examined using an RNA Nano 6,000 kit on a Bioanalyzer 2100 system (Life Technologies, Carlsbad, CA, USA).

For each sample, a total of 3 μg of RNA was loaded to generate the sequencing library using a NEBNext^®^ Ultra™ RNA library prep kit for Illumina^®^ (NEB, USA) following the manufacturer's instructions. The RNA was purified using an AMPure XP system, and the RNA quality was evaluated on an Agilent Bioanalyzer 2100 system. Gene clustering was performed using the TruSeq PE Cluster Kit v3-cBot-HS (Illumina, San Diego, CA, USA). Paired-end reads of 150 bp were generated for mRNA sequencing, and 50-bp single-end reads were generated for miRNA sequencing, using an Illumina HiSeq 2500 sequencer.

### 2.5. RNA sequencing data analysis

Clean reads were obtained from raw reads (FASTQ format) by removing adapter- or poly-N-containing reads and low-quality reads. The Q20, Q30, and GC contents were calculated. Clean reads were aligned with the reference genome (rat release-91) downloaded from the Ensembl database using Bowtie v2.2.3 (22) and TopHat v2.0.12 (23). The fragments per kilobase of transcript per million mapped reads of each transcript was calculated using Cuffdiff v2.1 (24). miRNA expression was compared with the expression of miRNA precursors and corresponding mature miRNAs in miRbase v22 (25) using miRDeep2 (26). Cuffdiff v2.1 was used to analyze the differentially expressed mRNA. The miRNA differential expression was analyzed using the DESeq R package. Genes with an adjusted *P*-value < 0.05 were identified as differentially expressed genes. The miRNA–mRNA interactions were predicted using RNAhybrid, PITA, and miRanda (27). Target genes of the differentially expressed miRNAs were subjected to Gene Ontology (GO) and Kyoto Encyclopedia of Genes and Genomes (KEGG) analysis using GOseq (28) and KEGG Orthology Based Annotation System (29).

### 2.6. Quantitative real-time PCR

Quantitative real-time (qRT)-PCR was conducted to measure the expression of differentially expressed miRNAs and mRNAs. For mRNA expression determination, complementary DNA was synthesized using SuperScript™ III SuperMix (Thermo Fisher Scientific, Waltham, MA, USA) according to the manufacturer's instructions. Primers for *Slc17a6, Mef2c, Fyn*, and *Slc25a22* were designed using the National Center for Biotechnology Information primer-blast (http://www.ncbi.nlm.nih.gov/tools/primer-blast/), and the sequences are summarized in Table 1. Glyceraldehyde 3-phosphate dehydrogenase was used as an internal control. PCR was performed using SYBR Power Plus Master Mix (Thermo Fisher Scientific). The conditions for real-time PCR were 95°C for 10 min, followed by 40 cycles of 95°C for 15 s, and 60°C for 60 s. Melting curve analysis was performed from 65 to 95°C in increments of 0.5°C. For miRNA expression determination, complementary DNA was synthesized using a TaqMan^®^ miRNA reverse transcription kit (Applied Biosystems, Foster City, CA, USA) following the manufacturer's protocol. PCR was performed using TaqMan^®^ miRNA Assays and TaqMan^®^ Universal PCR master mix (Applied Biosystems) on an Applied 7,500 device. Conditions for real-time PCR were 95°C for 10 min, followed by 50 cycles of 95°C for 15 s and 60°C for 60 s. Melting curve analysis was performed from 65 to 95°C in increments of 0.5°C. The miRNA primers are summarized in Table 2. U6 small nuclear RNA was used as an internal control. The mRNA or miRNA expression was quantified using the 2^−ΔΔCt^ method.

**Table 1:** Primers for quantitative real-time PCR.

**Primer name**	**Primer sequence**
*GAPDHqPCRF*	5′-CACCAGCATCACCCCATT-3′
*GAPDHqPCRR*	5′-CCATCAAGGACCCCTTCATT-3′
*Slc17a6qPCRF*	5′-TACGGTACCACCAAATCCTACGG-3′
*Slc17a6qPCRR*	5′-CTCGGTCCTTATAGGCGTACG-3′
*Mef2cqPCRF*	5′-AGCAGCAGCACCTACATAACAT-3′
*Mef2cqPCRR*	5′-TAGGAACTGCTACAGCTGCTCA-3′
*FynqPCRF*	5′-ATGGGCTGTGTGCAATGTAAG-3′
*FynqPCRR*	5′-GAAGCTGGGGTAGTGCTGAG-3′
*Slc25a22qPCRF*	5′-GCCAGCCAAGCTCATCAATG-3′
*Slc25a22qPCRR*	5′-GAGGCAGTCGGACATGCTC-3′

**Table 2:** MicroRNA primers for quantitative real-time PCR.

**MicroRNA**	**Primer sequence**
*miR-1224*	5′-CTCCACCTCCCCAGTCCTCAC-3′
*miR-137-3p*	5′-CTACGCGTATTCTTAAGCAATAA-3′
*miR-19b-3p*	5′-TCAGTTTTGCATGGATTTGCACA-3′
*miR-190a-5p*	5′-ACCTAATATATCAAACATATCA-3′
*miR-20a-5p*	5′-CTACCTGCACTATAAGCACTTTA-3′
*miR-211-5p*	5′-AGGCAAAGGATGACAAAGGGAA-3′
*miR-298-3p*	5′-AGCAGAGAGAAGGCTAGTTCCT-3′
*miR-30e-5p*	5′-CTTCCAGTCAAGGATGTTTACA-3′
*miR-335*	5′-ACATTTTTCGTTATTGCTCTTGA-3′
*miR-449a-5p*	5′-ACCAGCTAACAATACACTGCCA-3′
*miR-466c-5p*	5′-CATGTACATACACACATCACA-3′
*miR-494-3p*	5′-AGAGGTTTCCCGTGTATGTTTCA-3′
*miR-98-5p*	5′-AACAATACAACTTACTACCTCA-3′
*RNU6*	5′-TTGCGTGTCATCCTTGCGCAGG-3′

### 2.7. Statistical analysis

Data are expressed as mean ± standard deviation. Statistical analysis was performed using SPSS 25.0 (IBM, Armonk, NY USA). Graphs were generated using GraphPad Prism 9 (San Diego, CA, USA). The correlation between mRNA and miRNA was assessed using Pearson's correlation coefficient. Different groups were compared using a one-way ANOVA followed by a *t*-test of least significant difference. A *P*-value < 0.05 was considered statistically significant.

## 3. Results

### 3.1. Successful establishment of a post-traumatic epilepsy rat model

**Figure 1:**
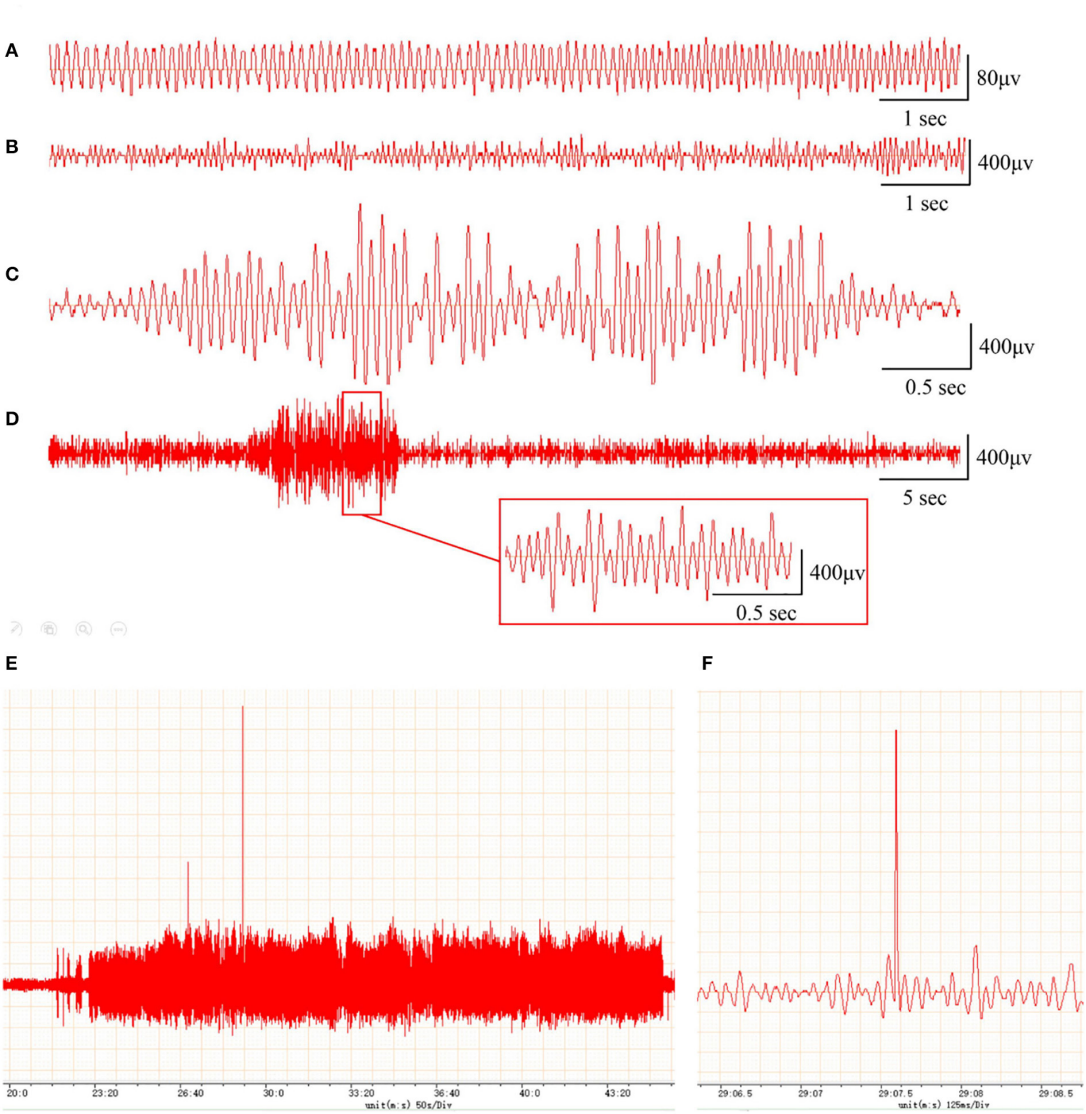
Rats with FeCl_2_ injection-induced post-traumatic epilepsy (PTE) showed epileptiform discharges on electroencephalogram (EEG). A total of 12 rats were randomly divided into sham and PTE groups (*n* = 6/group). Rats in PTE group were injected with 10-μl FeCl_2_ (0.4 mol/L, 1 μl/min) at right frontal cortex. Sham group underwent same procedures except for injection. A 1-h EEG, including first 15 min and blocks of 10 min at 5-min intervals, was recorded at 1 h before injection and at 1 h and 1, 7, and 30 days after injection. Representative EEGs are shown. **(A)** Normal EEG at 60 min before injection. Multiple spikes **(B)**, two- and three-phase sharp waves **(C)**, 10-s continuous abnormal discharges **(D)**, 20-min explosive abnormal discharges with occasional high-amplitude spikes **(E)**, and a sudden high-amplitude spike **(F)** were observed on day 30 after injection.

To assess whether the PTE rat model was successfully established, we examined behavioral seizures and EEGs in rats before and after FeCl_2_ injection. We found that, compared with sham rats, rats in the PTE group developed behavioral seizures after FeCl_2_ injection, including paroxysmal binocular immobility, staring, head nodding or wet-dog shaking, facial twitching, and generalized tonic–clonic seizures. Behavioral seizures occurred frequently within 3 days of the injection and declined thereafter until a regular behavioral seizure developed around 15 days after the injection. The total Racine's score of the PTE group was 810, with a mean value > 4 for each rat. In addition, compared with the EEG of the sham rats (frequency 5–10 Hz, amplitude < 200 μV; Figure 1A), the EEG of the PTE rats exhibited multiple epileptiform discharges, such as sharp-wave polyspikes (Figure 1B), waves (Figure 1C), spikes (Figures 1E, F), and continuous abnormal discharges (Figure 1D) with a maximum amplitude of 1,000 μV. Together, these results suggest that FeCl_2_ injection successfully induces PTE in rats.

### 3.2. Differentially expressed messenger RNAs and miRNAs in frontal lobes of post-traumatic epilepsy rats

**Figure 2:**
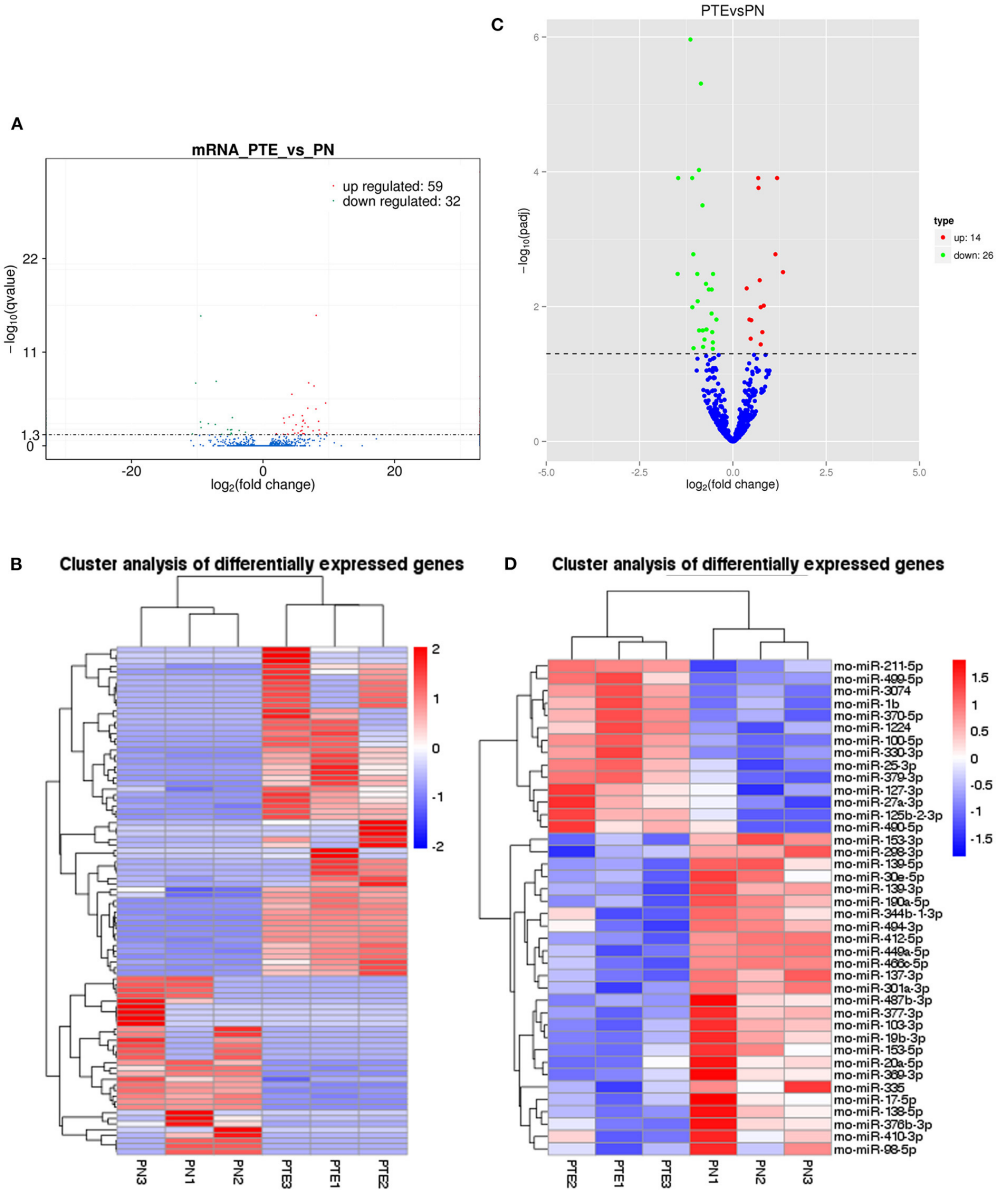
mRNA and miRNA transcriptome analysis in rat frontal lobe by RNA sequencing (RNA-Seq). **(A)** A volcano plot of mRNA sequence transcriptome data transcriptome data displays gene expression values of PTE rats compared with those of sham rats. Significantly differentially expressed genes (adjusted *P*-value <0.05) are highlighted in red (upregulated) or blue (downregulated). Non-differentially expressed genes are highlighted in green. *n* = 6. **(B)** Heat map of 59 significantly upregulated and 32 significantly downregulated mRNAs in PTE rats compared with sham rats. **(C)** A volcano plot of miRNA sequence transcriptome data displays gene expression values of PTE rats compared with those of sham rats. Significantly differentially expressed genes (adjusted *P*-value <0.05) are highlighted in red (upregulated) or blue (downregulated). Non-differentially expressed genes are highlighted in green. *n* = 6. **(D)** Heat map of 14 significantly upregulated and 26 significantly downregulated miRNAs in PTE rats compared with sham rats.

In this study, transcriptome sequencing was used to identify differentially expressed mRNAs and miRNAs in the frontal brain tissue of PTE rats. After comparing the mRNA and miRNA expression levels in the frontal brain tissue of rats in the PTE group and the normal control group, we found that 91 mRNAs and 40 miRNAs were abnormally expressed in the brain tissue of rats in the PTE group. According to Figures 2A, B, among the 91 mRNAs with abnormal expression, 59 mRNAs were upregulated (65%) and 32 mRNAs were downregulated (35%). Of these, eight differentially expressed mRNAs were associated with glutamatergic energy, six of which were upregulated and two of which were downregulated. According to Figures 2C, D, of the 40 miRNAs with abnormal expression, 14 were upregulated (35%) and 26 were downregulated (65%). The transcriptome sequencing results showed that multiple mRNAs and miRNAs were abnormally expressed in the brain tissue of rats with PTE, suggesting that mRNAs and miRNAs are involved in the occurrence and development of PTE and may play an important regulatory role.

### 3.3. Messenger RNAs by gene ontology annotation and kyoto Encyclopedia of Genes and Genomes pathway enrichment analysis

**Figure 3:**
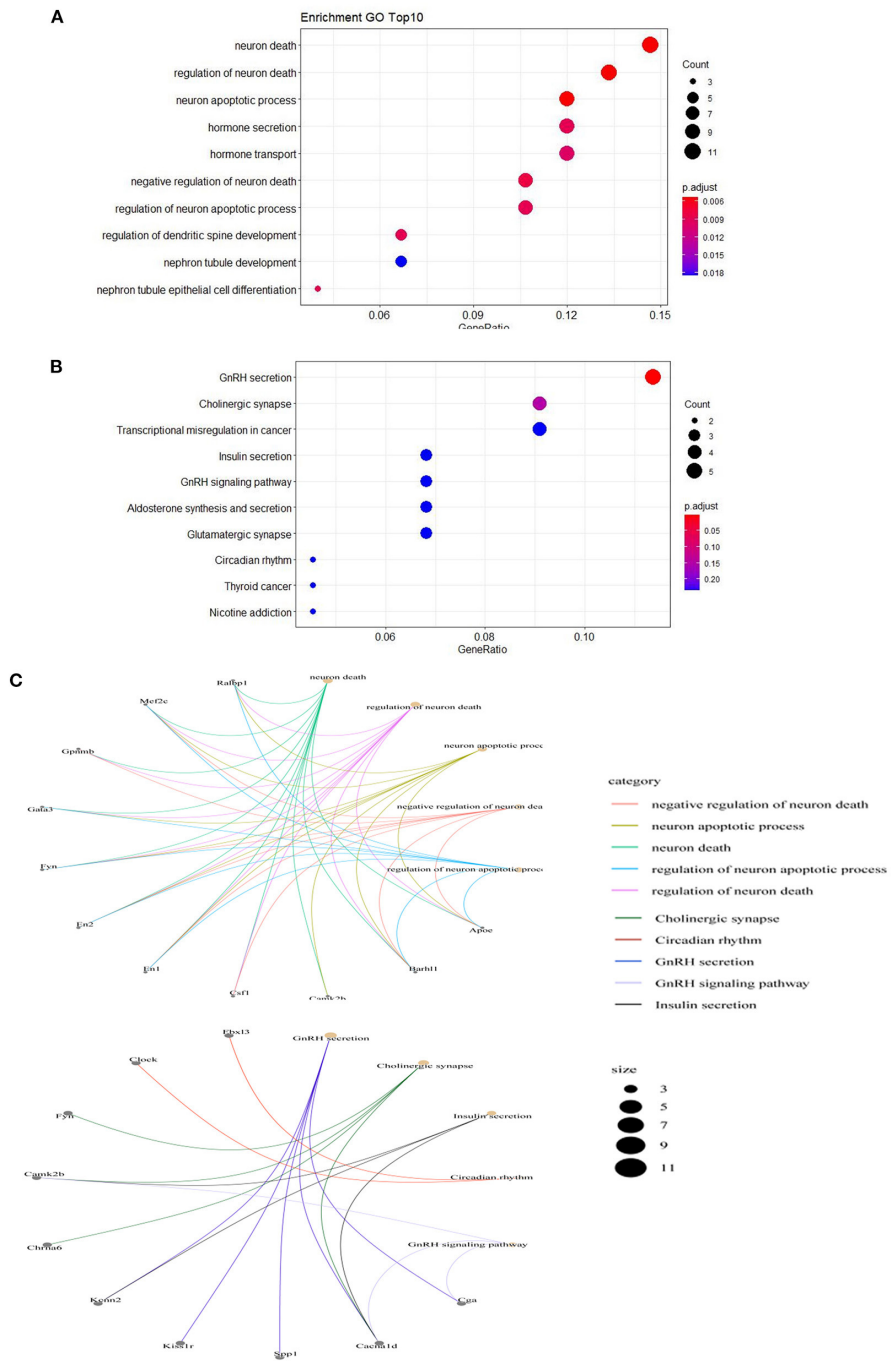
Gene Ontology annotation and Kyoto Encyclopedia of Genes and Genomes pathway analysis of differentially expressed mRNAs. **(A)** Top 10 Gene Ontology Annotation *P* < 0.05. **(B)** Top 10 Kyoto Encyclopedia of Genes and Genomes pathways *P* < 0.05. **(C)** The network diagram of relationship between enrichment function and gene inclusion.

To understand the function of the differentially expressed mRNAs in PTE, GO and KEGG enrichment analyses were performed on mRNAs with abnormal expression. Visualization of both the GO and KEGG enrichment analyses were performed with bubble diagrams (Figure 3A). The abscissa of the bubble plot is the ratio, the left ordinate is the GO term/metabolic pathway, the right ordinate is the *P*-value, and the size of the bubble indicates the number of genes. GO analyses showed that the functions of the 91 differential mRNAs dysregulated in PTE were mainly enriched in neuron death, regulation of neuron death, neuron process, hormone development, hormone development transport, negative regulation of neuron death, regulation of neuron process, and regulation of dendritic spine GO terms such as development. Thus, dysregulated mRNAs in PTE mainly participate in the processes of neuronal death and apoptosis. KEGG enrichment analyses (Figure 3B) showed that the 91 differential mRNAs dysregulated in PTE were mainly involved in metabolic pathways including GnRH development, cholinergic synapse, transcriptional misregulation in cancer, insulin secretion, the GnRH signaling pathway, and glutamatergic synapse. Figure 3C shows a network diagram of differential genes in PTE, showing the relationship between enrichment function and gene inclusion. According to Figure 3C, differentially expressed genes such as *Mef2c, Fyn, En1, En2, Aspa, Camk2b, Barh11*, and *Cacna1d* may participate in PTE in various ways.

### 3.4. Construction of miRNA–messenger RNA regulatory network

**Figure 4:**
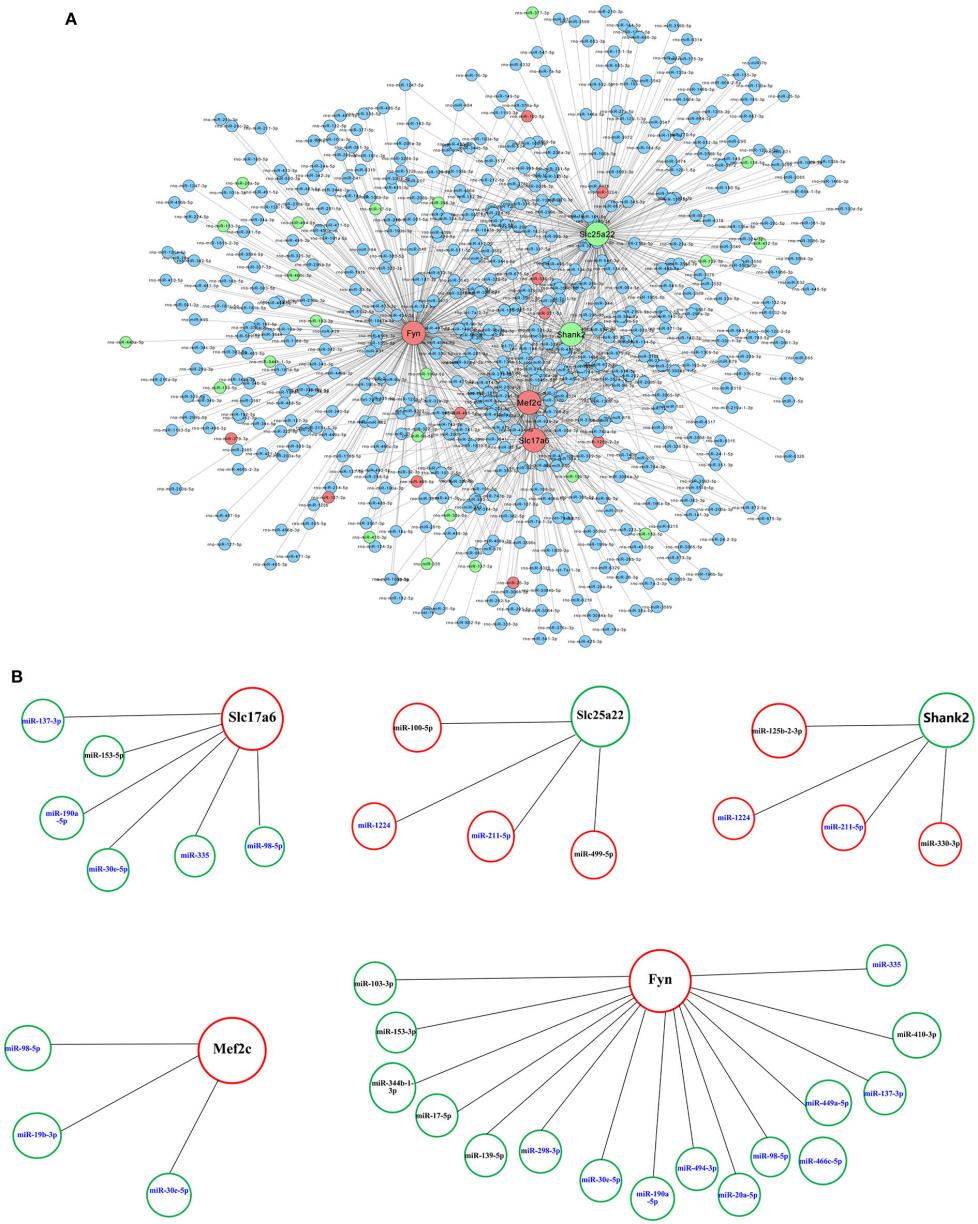
Prediction of microRNA–mRNA interaction. **(A)** Interaction network of microRNAs with 8 glutamatergic-related mRNAs. **(B)** Interaction network of 31 microRNAs with five glutamatergic-related mRNAs. Larger dots indicate mRNAs. Smaller dots indicated microRNAs. Red indicates upregulated mRNAs or microRNAs. Green indicated downregulated mRNAs or microRNAs. Blue indicates unchanged mRNAs or microRNAs.

Table 3 shows the differential mRNAs and functional annotations related to the glutamatergic system in PTE, with a total of eight dysregulated mRNAs related to the glutamatergic system. Among the eight mRNAs, six mRNAs were upregulated, namely *Slc17a6, Mef2c, Fyn, Tas1r2, Aspa*, and *Cacna1d*, and two mRNAs were downregulated, namely *Slc25a22* and *Shank2*. The targeting relationship prediction results showed that the dysregulation of eight mRNAs and 31 miRNAs related to the glutamatergic system was related to PTE. After statistical analysis, 33 pairs of miRNA–mRNA negative regulatory relationships related to the glutamatergic system in PTE were found, and the results are shown in Figures 4A, B. Because we did not find miRNAs targeting *Aspa, Cacna1d*, and *Tas1r2* differentially represented in PTE, the predictions for these mRNAs are not shown in the targeting relationship diagram. Further statistical analysis of the negative regulatory relationships between miRNA–mRNA from the miRNA perspective revealed that three downregulated miRNAs, miR-137-3p, miR-190a-5p, and miR-335, have negative regulatory relationships with *Slc17a6* and *Fyn*. Both miR-30e-5p and miR-98-5p have negative regulatory relationships with *Slc17a6, Mef2c*, and *Fyn*, and both miR-1224 and miR-211-5p have negative regulatory relationships with *Slc25a22* and *Shank2*.

**Table 3:** Differential mRNAs and functional annotations related to the glutamatergic system in PTE.

**Gene_ID**	**Gene_name**	**Gene_description**
ENSRNOG00000016147	Slc17a6	Glutamatergic synapse
ENSRNOG00000033134	Mef2c	Regulation of synaptic transmission, glutamatergic, synaptic transmission, glutamate receptor signaling pathway, regulation of glutamate receptor signaling pathway, regulation of N-methyl-D-aspartate selective glutamate receptor activity, regulation of alpha-amino-3-hydroxy-5-methyl-4-isoxazole propionate selective glutamate receptor activity
ENSRNOG00000000596	Fyn	Glutamate receptor binding, type 5 metabotropic glutamate receptor binding, G-protein coupled glutamate receptor binding, glutamate receptor signaling pathway, ionotropic glutamate receptor signaling pathway
ENSRNOG00000061876	Tas1r2	Glutamate metabolic process, proline catabolic process to glutamate, aspartate and glutamate metabolism
ENSRNOG00000019659	Aspa	Succinyl glutamate desuccinylase, aspartate and glutamate metabolism
ENSRNOG00000013147	Cacna1d	Glutamatergic synapse
ENSRNOG00000018450	Slc25a22	High-affinity glutamate transmembrane transporter activity, L-glutamate transmembrane transporter activity, L-glutamate transport
ENSRNOG00000050206	Shank2	Glutamatergic synapse

### 3.5. qRT-PCR validation and correlation evaluation of miRNA and glutamatergic messenger RNAs

**Figure 5:**
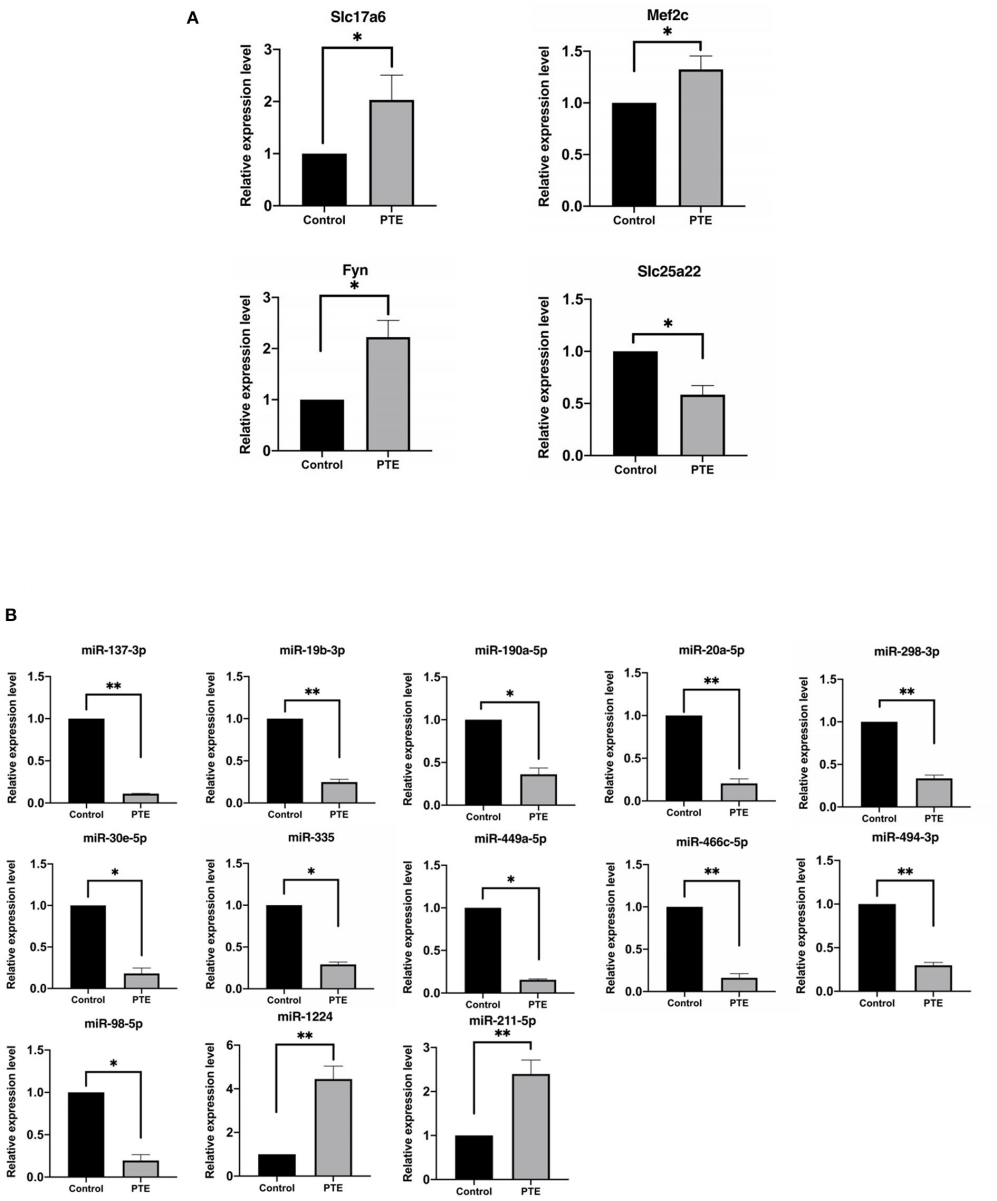
Quantitative real-time PCR verification of differentially expressed glutamatergic-related mRNAs and microRNAs. *P < 0.05, **P < 0.01 vs. sham group. **(A)** The expression of verified mRNAs (Slc17a6, Mef2c, and Fyn) in PTE group was significantly increased compared with the control group, and the expression of verified mRNAs (Slc25a22) in PTE group was significantly decreased compared with the control group. **(B)** The expression of verified microRNAs (miR-137-3p, miR-19b-3p, miR-190a-5p, miR-20a-5p, miR-298-3p, miR-30e-5p, miR-335, miR-449a-5p, miR-466c-5p, miR-494-3p, miR-98-5p) in the PTE group was significantly decreased compared with the control group, and the expression of verified mRNAs (miR-1224 and miR-211-5p) in PTE group was significantly increased compared with the control group.

To verify the accuracy of the above transcriptome sequencing results, in combination with literature mining of miRNA function and differential multiples, we applied qRT-PCR to verify the expression differences of the above five mRNAs and 13 mRNAs with targeting relationships. qRT-PCR verification results of the mRNA (Figure 5A, Table 4) showed that, compared with the normal control group, the expression levels of *Slc17a6, Mef2c*, and *Fyn* in the PTE experimental group were significantly upregulated, and the expression level of *Slc25a22* was significantly downregulated, and the differences were statistically significant (*P* < 0.05). This was consistent with the transcriptome sequencing results. However, no significant difference in *Shank2* expression was detected. The qRT-PCR verification results for the miRNAs (Figure 5B, Table 4) showed that, compared with the normal control group, in the PTE group, the expression levels of miR-137-3p, miR-19b-3p, miR-190a-5p, miR-20a-5p, miR-298-3p, miR-30e-5p, miR-335, miR-449a-5p, miR-466c-5p, miR-494-3p, and miR-98-5p were significantly upregulated, while the expression levels of miR-1224 and miR-211-5p were significantly downregulated, and the differences were statistically significant (*P* < 0.05). This was consistent with the results of the transcriptome sequencing.

**Table 4:** Relative expression level of mRNA and miRNA detected in PTE experimental group.

**Name of mRNA and miRNA**		**Relative expression level (mean ±SD)**	***P*** **-value**	**Regulation trend**
mRNA	Slc17a6	2.03 ± 0.48	<0.05	↑
	Mef2c	1.32 ± 0.13	<0.05	↑
	Fyn	2.22 ± 0.33	<0.05	↑
	Slc25a22	0.58 ± 0.09	<0.05	↓
miRNA	miR-137-3p	0.11 ± 0.00	<0.01	↓
	miR-19b-3p	0.25 ± 0.03	<0.01	↓
	miR-190a-5p	0.36 ± 0.07	<0.05	↓
	miR-20a-5p	0.21 ± 0.05	<0.01	↓
	miR-298-3p	0.34 ± 0.04	<0.01	↓
	miR-30e-5p	0.21 ± 0.06	<0.05	↓
	miR-335	0.29 ± 0.03	<0.05	↓
	miR-449a-5p	0.20 ± 0.07	<0.05	↓
	miR-466c-5p	0.16 ± 0.05	<0.01	↓
	miR-494-3p	0.30 ± 0.03	<0.01	↓
	miR-98-5p	0.20 ± 0.07	<0.05	↓
	miR-1224	4.45 ± 0.6	<0.01	↑
	miR-211-5p	2.40 ± 0.31	<0.01	↑

**Figure 6:**
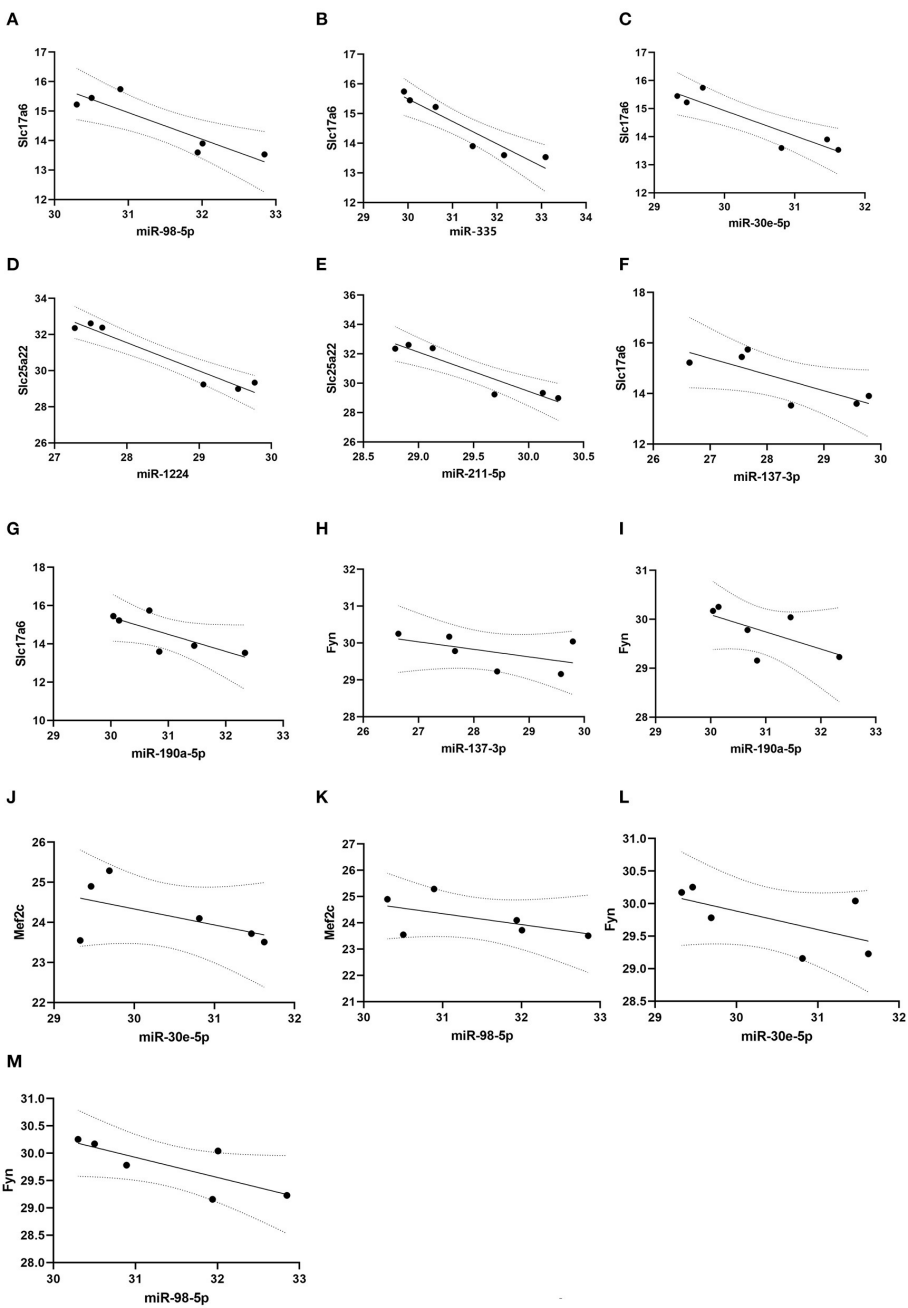
Correlation analysis. Pearson correlation analysis was conducted to evaluate the correlation between microRNAs and glutamatergic-related mRNAs. **(A–C)** miR-98-5p, miR-335 and miR-30e-5p expression were significantly and negatively correlated with Slc17a6 expression; **(D, E)** miR-1224 and miR-211-5p expression were negatively correlated with Slc25a22 expression. **(F–I)** miR-137-3p and miR-190a-5p expression were no significantly and negatively correlated with Slc17a6 and Fyn expression. **(J–M)** miR-30e-5p and miR-98-5p expression were no significantly and negatively correlated with Mef2c and Fyn expression.

Pearson correlation analysis showed that expressions of miR-98-5p (*r* = −0.90, *P* < 0.05), miR-335 (*r* = −0.95, *P* < 0.05), and miR-30e-5p (*r* = −0.92, *P* < 0.05) were significantly negatively correlated with *Slc17a6* expression (Figures 6A–C); and miR-1224 (*r* = −0.97, *P* < 0.05) and miR-211-5p (*r* = −0.94, *P* < 0.01) were negatively correlated with *Slc25a22* expression (Figures 6D, E). However, we did not observe significant negative correlation between miR-137-3p (*r* = −0.78, *P* > 0.05) /miR-190a-5p (*r* = −0.77, *P* > 0.05) and *Slc17a6*/*Fyn* expression; and miR-30e-5p (*r* = −0.55, *P* > 0.05) and miR-98-5p (*r* = −0.55, *P* > 0.05) were significantly negatively correlated with *Mef2c* and *Fyn* (Figures 6F–M).

## 4. Discussion

PTE is an acquired form of epilepsy caused by trauma. Studies have confirmed that increased glutamate responses modulate neuronal microcircuits after TBI, which correlates with increased seizure-like activity near the injury site (30). Considering the important role of glutamatergic dysregulation in the development of PTE, we used transcriptome sequencing technology, miRNA–mRNA regulatory network construction, GO and KEGG enrichment analyses, and other bioinformatics methods to analyze the mRNAs and miRNAs with abnormal expression in PTE to understand the molecular mechanism of the glutamatergic system in PTE. Based on the predicted targeting relationships, qRT-PCR was performed to validate the five mRNAs with anomalous expression and associated glutamatergic energy and the 13 mRNAs targeted by them. The results showed that there were indeed abnormal expression and regulation of miRNA–mRNA related to the glutamatergic system in the PTE model, which is very likely to play an important regulatory role in the occurrence of PTE. These abnormally expressed RNA molecules are likely to be involved in the pathogenesis of PTE, which may not only become a new diagnostic biomarker of PTE in the future but also a potential therapeutic target for clinical treatment of PTE. Considering that the changes to miRNAs and mRNAs after TBI may affect the development of PTE, combined with the neuropathological changes after TBI, we will discuss the mechanism of abnormal miRNA and mRNA expression in the PTE model involved in the development of PTE.

After TBI, Ca^2+^ channels are activated, and a large amount of Ca^2+^ influx induces neuronal apoptosis (31). However, massive influx of Ca^2+^ simultaneously causes rapid depolarization, and abnormal excitation of nerve cells directly leads to seizures (32). Therefore, the neuronal apoptosis induced by massive Ca^2+^ influx after TBI leads to the decrease of inhibitory neurotransmission, and the increase of excitatory neurotransmission caused by Ca^2+^ influx can directly cause the neuronal excitatory/inhibitory (E/I) imbalance. This imbalance is likely to be the mechanism of PTE.

The results of this study suggest that expression of the *Mef2c* gene is upregulated in the frontal brain tissue of PTE rats. Expression of the MEF2C transcription factor triggers upregulation of NMDA receptor subunit 1 (NR1) (33). However, NMDA receptors are unique dual-gated channels, activation of which can lead to massive influx of Ca^2+^ and rapid depolarization of neuronal cells, which can directly lead to seizures (32). Combined with the phenomenon of massive Ca^2+^ influx after TBI, we hypothesized that after TBI, the expression of the *Mef2c* gene is upregulated and the NMDA receptor is activated, leading to massive Ca^2+^ influx, which is then involved in the occurrence of PTE. However, TBI and microglia-mediated inflammation inhibit the expression of more than 200 cortical neuron genes, including MEF2C, and MEF2C-HET mice exhibit excitatory/inhibitory (E/I) imbalance resulting from decreased inhibitory neurotransmission and increased excitatory neurotransmission (34, 35). These findings suggest that *Mef2c* may bidirectionally regulate neuronal death and promote apoptosis after TBI. Therefore, although our experimental results confirmed that the upregulation of *Mef2c* gene expression in the PTE model could explain the mechanism of PTE through neuronal excitatory/inhibitory (E/I) imbalance, microglia may inhibit the expression of *Mef2c* because microglia activation is a common feature of TBI and PTE. Therefore, further studies are needed to clarify the mechanism of *Mef2c* in the development of PTE (36).

Caspase-3 colocalizes with tau accumulation in CCI rats, indicating a possible correlation between apoptosis and tau in the chronic phase of TBI (37). A decrease in tau levels is accompanied by a decrease in NMDA receptor-dependent Ca^2+^ influx, which leads to diminished neuroexcitoxic effects, and thus abnormal tau accumulation induced by TBI may also be associated with the development of PTE (38). Our experimental results show that the expression of *Fyn* is upregulated in the PTE model. The FYN protein is one of the nine members of the Src family of non-receptor tyrosine kinases (SFK), which can phosphorylate metabolic and ionic glutamate receptors and regulate their subcellular distribution and function, thus regulating glutamatergic synaptic signaling (39–41). However, the phosphorylation of tau at tyrosine 18 (pY18) mediated by FYN protein determines the Ca^2+^ influx and the degree of nerve excitation associated with tau protein. Therefore, upregulation of *Fyn* may lead to changes in FYN protein expression and the consequent abnormal accumulation of tau protein, which is involved in the development of PTE (38). However, some scholars believe that tau plays an independent role from FYN in regulating the Ca^2+^ response (42). Therefore, although our experiments confirm the upregulation of *Fyn* gene expression in the PTE model, whether *Fyn* is involved in the occurrence of PTE by mediating the tau protein remains to be confirmed by further studies.

Furthermore, astrocytes play an important role in post-injury brain repair, and astrocyte-mediated mechanisms involved in synaptic development may play an important role in neuronal injury-induced synaptic remodeling. However, intermittent reduction or loss of neuronal excitatory input after TBI can induce production of TNFα by reactive astrocytes, and overexpression of TNFα is associated with the development of epilepsy (43). The expression levels of FYN protein and phosphorylated extracellular signal-regulated kinase ERK1/2 (p-ERK1/2) were significantly increased in epilepsy models, and the expression of p-ERK1/2 was significantly decreased when FYN expression was downregulated, suggesting that the activation of the ERK1/2 signaling pathway may be regulated by *Fyn* expression (44). ERK1/2 signaling is involved in the activation and proliferation of astrocytes after TBI, which can affect the occurrence and recurrence of epilepsy (45). Therefore, in addition to the induction of PTE through tau, *Fyn* may also trigger astrocyte activation and proliferation through activation of the ERK1/2 signaling pathway, thereby enhancing susceptibility to PTE after TBI.

With the exception of *Mef2c* and *Fyn*, our experimental results show that *Slc17a6* expression is upregulated while *Slc25a22* expression is downregulated. *Slc17a6* encodes the VGLUT2 protein, a glutamate transporter that plays a key role in regulating the release of glutamate neurotransmitters. Changes in VGLUT2 protein levels can affect glutamate signaling (46). Therefore, upregulation of VGLUT2 protein leads to abnormal neuronal discharge, which may be an important mechanism for the occurrence and development of PTE. *Slc25a22 (*solute carrier family protein 25 member 22) is a mitochondrial glutamate transporter that is mainly expressed in astrocytes. *Slc25a22* mRNA and protein expression are significantly upregulated in tumor tissues and cell lines of gallbladder cancer, and downregulation of *Slc25a22* suppresses tumor cell migration and proliferation and promotes apoptosis. This apoptosis-promoting mechanism of *Slc25a22* may be achieved by downregulating the MAPK/ERK pathway (47). At the same time, downregulation of *Slc25a22* reduces glutamate catabolism in astrocytes and leads to accumulation of glutamate in cells. Therefore, the accumulation of glutamate triggered by *Slc25a22* downregulation may also be the initiating factor of abnormal neuronal excitation (48).

miRNAs may target multiple mRNAs individually, leading to multiple biological mechanisms. Combined with the existing results, we predict that the abnormal expression of some miRNAs may be involved in the occurrence of PTE by promoting apoptosis. The expression of miR-137-3p and miR-335 are downregulated in the PTE model used in this study. Studies have confirmed that miR-137-3p is significantly downregulated in both prostate cancer and colorectal cancer and can target specific proteins to inhibit tumor growth and metastasis and promote apoptosis (49). In addition, miR-137-3p targets GRIN2A, a target of NMDA receptors. Therefore, miR-137-3p may also be involved in PTE development by affecting NMDA receptors in addition to apoptosis (50). miR-335 is a classical tumor suppressor, and its expression level is significantly upregulated in serum of TBI patients. miR-335 is abnormally expressed in both TBI and PTE, suggesting that it is involved in the development of epilepsy after TBI (51). These findings suggest that miR-137-3p and miR-335 may be involved in the development of PTE by promoting apoptosis. Aside from miR-137-3p and miR-335, our results suggest that miR-1224 is also upregulated. miR-1224 has been reported to be abnormally expressed in a variety of tumors, and its overexpression can significantly inhibit the proliferation and metastasis of cancer cells and increase their apoptosis, so it is likely that miR-1224 is also involved in the development of PTE in the same way (52, 53).

In addition to the mechanisms that promote apoptosis, some abnormally expressed miRNAs may participate in the development of PTE by promoting neuroinflammation and inhibiting apoptosis. Our study shows that miR-30e-5p is downregulated and miR-211-5p is upregulated in the PTE model. In the cardiomyocyte hypoxia model, miR-30e-5p expression was significantly downregulated, and miR-30e-5p overexpression was found to inhibit inflammation by inhibiting the expression of target genes, thereby alleviating myocardial injury (54). miR-30e-5p is overexpressed in acute kidney injury models and directly targets the *Beclin1* gene, a key regulator of autophagy, to inhibit autophagy and induce apoptosis (55). Thus, the downregulation of miR-30e-5p may be involved in the occurrence of PTE through the above two mechanisms. It should be noted that unlike cardiac or brain tissue, miR-30e-5p expression is upregulated in saliva and cerebrospinal fluid after TBI, but the exact mechanism is unknown. TBI is the basis of PTE damage, so the mechanism of the involvement of miR-30e-5p in the development of PTE needs to be further studied (56). In the rat middle cerebral artery blockade and reperfusion model, miR-211-5p expression was decreased in the cerebral cortex of rats, while miR-211-5p overexpression significantly reduced cell apoptosis and lactate dehydrogenase (LDH) release rate, thereby improving cell viability (57). It has also been demonstrated that miR-211-5p expression is upregulated in the cerebral cortex of rats with a chronic inflammatory endophenotype induced by TBI (58). In combination with the upregulation of miR-211-5p expression in the PTE model, we hypothesize that miR-211-5p may be involved in the occurrence of PTE by inhibiting apoptosis or enhancing LDH release.

The involvement of miR-98-5p in PTE may be more complex than other miRNAs. miR-98-5p is downregulated in the mouse middle cerebral artery occlusion reperfusion model (MCAO/R) and the serum of stroke patients. Upregulation of miR-98-5p can alleviate the symptoms of cerebral ischemia in MCAO/R mice and reduce oxidative stress injury by inhibiting the production of reactive oxygen species (ROS). Apoptosis was also inhibited by reducing protein kinase 1 (DAPK1), B-cell lymphoma/leukemia-2 (Bcl-2)-associated X protein (BAX), and cleaved caspase-3 levels (59). In addition, miR-98-5p can negatively regulate the expression of IL-6 and is related to the inflammatory response (60). Moreover, tumor-related studies have also confirmed that miR-98-5p regulates cell proliferation and apoptosis (61). This all suggests that miR-98-5p may be involved in PTE through mechanisms such as inhibition of oxidative stress, altered cell proliferation, apoptosis, and inhibition of the inflammatory response.

In addition to screening PTE differential genes, this study also constructed a miRNA–mRNA regulatory network related to the PTE glutamatergic system based on transcriptome detection results, and performed qRT-PCR experiments to validate some miRNA–mRNA negative regulatory pairs in the regulatory network. It is possible that miR-137a-3p, miR-190a-5p, and miR-335 may be involved in the occurrence of PTE by modulating the expression of *Slc17a6* and *Fyn*. Based on the prediction and validation results of the targeting relationship, it is possible that miR-137a-3p, miR-190a-5p, and miR-335 may be involved in the occurrence of PTE. Similarly, miR-30e-5p and miR-98-5p may be involved in the development of PTE by modulating the expression of *Slc17a6, Mef2c*, and *Fyn*, and miR-19b-3p may be involved in the development of PTE by upregulating *Mef2c*. miR-20a-5p, miR-298-3p, miR-449a-5p, miR-466c-5p, and miR-494-3p may be involved in the occurrence of PTE by upregulating *Fyn*. The expression of miR-1224 and miR-211-5p in the PTE model is significantly upregulated, and they may be involved in the occurrence of PTE by downregulating the expression of *Slc25a22*. Need to add that, miR-19b-3p, miR-20a-5p, miR-449a-5p and miR-494-3p are related to apoptosis (62–65). And it is noteworthy that miR-298-3p and miR-466c-5p are rarely reported (66, 67). Although their functions are poorly understood at present, they are promising diagnostic markers of PTE as newly discovered regulatory factors.

Statistical analysis showed that miR-98-5p (*r* = −0.90, *P* < 0.05), miR-335 (*r* = −0.95, *P* < 0.05), and miR-30e-5p (*r* = −0.92, *P* < 0.05) were negatively correlated with *Slc17a6* expression, and miR-1224 (*r* = −0.97, *P* < 0.05) and miR-211-5p (*r* = −0.94, *P* < 0.01) were negatively correlated with *Slc25a22* expression. Of the above predicted regulatory pairs, only the direct targeting relationship between miR-190a-5p and *Slc17a6* has been confirmed by previous studies. Whether other regulatory relationships have direct targeted regulatory relationships and how specific regulatory mechanisms work are still at the prediction stage and need to be validated by repeated *in vivo* experiments in cells and animals.

Our study has some limitations, including lacks of verification regarding the physical bindings between miRNAs and mRNAs, the miRNA function and pathway analysis results associated with mRNA, the record of EEG for 24 h continuously for 30 days, and small sample size. In addition, about the experimental model, of the existing chemicals used to induce PTE, FeCl_2_ has been extensively practiced and accepted as the most successful chemical stimulator for PTE (68, 69). This is mainly because during the formation of PTE, hemosiderin plays an important role. Previous studies found that extravasation and dissolution of red blood cell and deposition of hemosiderin in neural network (CNN) occur following TBI, which are typical symptoms of TBI and closely related to epilepsy (70). As a result of trauma, subarachnoid hemorrhage and cerebral parenchymal hemorrhage often because blood accumulated in the cortical tissue, which leads to high risk of seizures. Animal experiments indicated that the epileptic effect of Fe ions is related to its redox reactions (71). In addition, because the process of producing epileptic susceptibility of FPI model is long (that is, several months after injury), and the generation of controlled cortical impact (CCI) model requires complex technical equipment, both methods have shortcomings (2). Therefore, we used FeCl_2_ model due to multiple factors. However, it is not clear whether the differences in the models used so far affect the results. These limitations will be addressed in future studies.

To sum up, in this study, we found glutamatergic-related mRNAs of *Slc17a6, Mef2c, Fyn*, and *Slc25a22*, as well as miR-137-3p, miR-190a-5p, miR-335, miR-30e-5p, miR-98-5p, miR-19b-3p, miR-20a-5p, miR-298-3p, miR-449a-5p, miR-466c-5p, miR-494-3p, miR-1224, and miR-211-5p. miR-98-5p (*r* = −0.90, *P* < 0.05), miR-335 (*r* = −0.95, *P* < 0.05), and miR-30e-5p (*r* = −0.92, *P* < 0.05) expression were negatively correlated with *Slc17a6* expression, while miR-1224 (*r* = −0.97, *P* < 0.05) and miR-211-5p (*r* = −0.94, *P* < 0.01) were negatively correlated with *Slc25a22* expression. We analyzed the association between abnormally expressed genes and GO and KEGG enrichment analyses to provide insights into the molecular mechanisms of glutamatergic enrichment in PTE. Our findings suggest that these miRNAs and miRNA-regulated alterations in glutamatergic mRNA expression are involved in the development of PTE, providing potential diagnostic biomarkers and therapeutic targets for the treatment of PTE.

## Data availability statement

The data presented in the study are deposited in the NCBI website repository, accessible with the following link, the Bioproject ID is PRJNA667324 (http://www.ncbi.nlm.nih.gov/bioproject/667324).

## Ethics statement

The animal study was reviewed and approved by the Animal Research Ethics Committee of the Institute of Evidence Science, China University of Political Science and Law.

## Author contributions

TY and JM conceived and designed the study. XZ and YM analyzed the data and wrote the initial draft of the manuscript. FZ, MZ, and DZ conducted the experiments and collected the data. XW contributed to refining the ideas. All authors were involved in revising the manuscript.
